# Feeding on dispersed vs. aggregated particles: The effect of zooplankton feeding behavior on vertical flux

**DOI:** 10.1371/journal.pone.0177958

**Published:** 2017-05-17

**Authors:** Marja Koski, Julia Boutorh, Christina de la Rocha

**Affiliations:** 1 National Institute for Aquatic Resources, Technical University of Denmark, Kemitorvet, Building 202, Lyngby, Denmark; 2 CNRS UMR 6539, Institut Universitaire Européen de la Mer, Université de Bretagne Occidentale, Technôpole Brest-Iroise, Place Nicholas Copernic, Plouzané, France; University of Connecticut, UNITED STATES

## Abstract

Zooplankton feeding activity is hypothesized to attenuate the downward flux of elements in the ocean. We investigated whether the zooplankton community composition could influence the flux attenuation, due to the differences of feeding modes (feeding on dispersed vs. aggregated particles) and of metabolic rates. We fed 5 copepod species—three calanoid, one harpacticoid and one poecilamastoid–microplankton food, in either dispersed or aggregated form and measured rates of respiration, fecal pellet production and egg production. Calanoid copepods were able to feed only on dispersed food; when their food was introduced as aggregates, their pellet production and respiration rates decreased to rates observed for starved individuals. In contrast, harpacticoids and the poecilamastoid copepod *Oncaea* spp. were able to feed only when the food was in the form of aggregates. The sum of copepod respiration, pellet production and egg production rates was equivalent to a daily minimum carbon demand of ca. 10% body weight^-1^ for all non-feeding copepods; the carbon demand of calanoids feeding on dispersed food was 2–3 times greater, and the carbon demand of harpacticoids and *Oncaea* spp. feeding on aggregates was >7 times greater, than the resting rates. The zooplankton species composition combined with the type of available food strongly influences the calculated carbon demand of a copepod community, and thus also the attenuation of vertical carbon flux.

## Introduction

The efficiency of the biological carbon pump is determined by attenuation of vertical carbon flux in, and below, the photic zone. Although traditionally it has been assumed that the flux attenuation is relatively constant irrespective of location [1], recent measurements have shown large variation in vertical flux and its attenuation with season, sampling location and even day [2, 3]. This variation is assumed to result from the differences in quality and quantity of sinking particles and changes in bacterial and zooplankton activity [4]. While the rate of marine snow degradation by bacteria appears to be a relatively constant 0.13 d^-1^ [5], total zooplankton degradation rate is likely to be much more variable as a result of differences in population dynamics, ecophysiology and behavior among areas, seasons and zooplankton species [6, 7].

Zooplankton exhibit a range of feeding modes. Most calanoid copepods are assumed to feed primarily by producing a feeding current [8], and appear to have a preferred predator to prey size ratio of ca. 20:1 [9]. Cyclopoids such as *Oithona* spp. are typically ambush feeders, and prefer motile prey [10]. Yet, some zooplankton species are specialized in feeding on sinking particles [11]. For instance, some copepod species such as pelagic harpacticoid *Microsetella norvegica* are not able to feed on small dispersed particles or micro-organisms, but rather need to attach to a particle to feed on its surface, in a manner similar to feeding of benthic organisms on fallen prey [12]. *M*. *norvegica*, as well as other harpacticoids and the poecilamastoid copepod *Oncaea* spp., have been frequently observed on marine snow particles [13, 14], suggesting that sinking aggregates are their primary food source [15, 16]. Some calanoid species have also been observed to feed on marine snow [17, 18, 19] and calculations of the energetic demand of mid-water copepods suggest that sinking particles form a large fraction of their diets [19, 20].

Besides differing in feeding mode, copepod species also differ in ecophysiology and in vertical migration behavior. For example, large calanoids feeding on diatoms egest a relatively large proportion of their food as large, fast sinking fecal pellets [21, 22, 23], while small copepod species such as *Oithona* sp. or *Microsetella norvegica* egest small slowly sinking pellets which are more likely to be degraded before sinking out of the surface layer [24].Similarly, copepod respiration rates vary and are usually related to temperature and body size [25, 26], feeding [27, 28] and swimming behavior [29]. Also vertical migration behavior is different between species, life-stages and e.g., seasons: While large calanoids perform dial vertical migration of several hundreds of meters in some regions [30], small copepods can stay in the same water layer during day and night. Thus, the effect of zooplankton on attenuation of vertical carbon flux should depend on species composition: a large vertically migrating calanoid copepod which feeds at a high rate on small phytoplankton cells in the euphotic zone should have a different effect than a small marine snow colonizing harpacticoid residing permanently below the euphotic zone.

The estimated energetic demand of heterotrophic organisms in mid-water is frequently larger than their estimated food availability (amount of carbon reaching the depth of their occurrence) [4, 31, 32]. The mid-water carbon budgets have typically been made for zooplankton populations resident in the twilight zone, using size-specific respiration rates of calanoid copepods [25], and assuming that respiration converted to carbon represents their minimum carbon demand [4, 32]. We investigated whether the zooplankton community composition could influence the carbon budgets, due to the differences of feeding modes (feeding on dispersed vs. aggregated particles) and of metabolic rates. We hypothesized that calanoid copepods depend on dispersed particles to meet their metabolic needs whereas poecilamastoid and harpacticoids copepods depend on aggregated food. Therefore, differences in feeding and metabolic rates between non-aggregate and aggregate feeders influence the short-term carbon demand of the zooplankton community. Our results suggest that zooplankton species composition combined with the type of the available food can strongly influence the carbon demand of a copepod community, and therefore also the attenuation of the vertical carbon flux.

## Materials and methods

Respiration, fecal pellet production and egg production rates of five copepod species were investigated by offering copepods either dispersed food or aggregated food. The food originated from a mesocosm with added nutrients, some including and some without silica (see below), which induced phytoplankton communities dominated by diatoms and flagellates, respectively. The water collected from the mesocosms was offered to copepods either directly (dispersed treatments) or after rotating in a rolling table (aggregated treatments). Individuals of five common copepod species collected from the study area were used in experiments: calanoid copepods *Temora longicornis*, *Acartia* sp. and *Centropages* sp., the poecilamastoid copepod *Oncaea* spp., and an unidentified harpacticoid copepod species.

### Mesocosm, copepods and food suspension

The experiments were run as part of a larger mesocosm experiment, conducted in August 2012 in Sletvig Field Station (Norwegian University of Science and Technology), in Bay of Hopavågen (63° 36’ N, 9° 33’ E)—a semi-enclosed marine lagoon. In total 12 mesocosms, each consisting of a 9 m^3^ polyethylene bag (diameter ca. 1 m, depth 9 m), were filled with lagoon water and had their concentrations enhanced by adding daily either a combination of 0.22 μM sodium nitrate and 0.014 μM sodium phosphate (NP, mesocosm #9) or the NP addition plus 0.22 μM sodium metasilicate (NPSi, mesocosm #1). The experiments described here were conducted between 15^th^ and 23^rd^ August, around the time when the algae biomass in all mesocosms peaked. Variations in temperature and salinity were low; 13.2–13.5°C and 31.5–32 ppm, respectively (Moriceau et al.; in prep).

Copepods for the experiments were collected from the lagoon by making repeated horizontal and vertical net hauls using a 90 μm plankton net with an opening of 0.25 m^2^ and having a non-filtering cod end. Horizontal hauls were collected by dragging the plankton net few meters below the surface behind a small motor boat, while vertical hauls were collected by slowly lifting the net by hand from ca. 10 m to the surface. The cod ends were immediately diluted into 30-litre buckets filled with surface water and brought to the laboratory.

The mesocosm water used for the feeding experiments was collected at 7 am using a 1.5 m long tube sampler lowered to collect an integrated sample from different water depths. Water samples were kept in 20-L carboys at close to *in situ* temperature until used in experiments a few hours later. Mesocosm water was filtered through a 180 μm net to remove mesozooplankton. Some of the mesocosm water was fed to copepods either directly so that the potential food particles were dispersed in the water (disp); some of the water was incubated in a rotating cylinder to form the food particles into aggregates [33], also known as marine snow (aggr; Table 1). The rolling tanks consisted of slowly rotating (1 RPM) 5-l Plexiglas cylinders kept in dark and in temperature of 18°C. After minimum of 12-h, all aggregates were collected from the tanks using a large-mouthed pipette under a stereo microscope. The aggregates were then re-suspended into the same volume of GF/F filtered seawater from which they originated. We assumed that this way the food concentration in dispersed and aggregated treatments would be approximately the same, although the concentrations in aggregate treatments would always be somewhat lower than in the dispersed treatments.

**Table 1:** List of the experiments, origin of the incubation water (date and number of the mesocosm; MK) and number of replicate egg and fecal pellet production and respiration experiments conducted with each copepod species. NP and NPSi refer to the mesocosms trials, respectively without and with added Si. C (*Centropages* sp.), T (*Temora longicornis*), A (*Acartia* sp.), H (harpacticoid sp.) and O (*Oncaea* spp.). (FW) indicates filtered seawater control with starved copepods. (Blank) No experiments.

**Experiment**	**Date**	**MK**	**Species and measurements (# of replicates)**		
			Egg production	Pellet production	Respiration
**NP Disp 1**	**16–17.8.**	**9**	**C(3), T(3), A(3)**	**C(3), T(3), A(3), H(3)**	**C(3), T(2), A(2), H(2)**
**NP Disp 2**	**21–22.8.**	**9**	**C(3), T(1), A(3)**	**C(3), T(1), A(3), H(2), O(3)**	**C(3), T(2), A(2), H(3)**
**NPSi Disp 1**	**16–17.8.**	**1**	**C(3), T(3), A(3)**	**C(3), T(3), A(3), H(3)**	**T(3), A(2), H(3), O(3)**
**NPSi Disp 2**	**21–22.8.**	**1**	**C(2), T(3), A(3)**	**C(2), T(3), A(3), O(3), H(3)**	**T(2)**
**NP Aggr 1**	**18–19.8.**	**9**	**C(1), T(2), A(3)**	**C(1), T(2), A(3)**	**C(1), T(2), A(2), H(2),O(3)**
**NP Aggr 2**	**22–23.8.**	**9**	**C(3), T(3)**	**C(3), T(3), H(3), O(2)**	**C(2), T(1), H(1), O(3)**
**NPSi Aggr 1**	**18–19.8.**	**1**	**C(1), T(3), A(2)**	**C(1), T(3), A(2)**	**T(2), H(3), O(3)**
**NPSi Aggr 2**	**22–23.8.**	**1**	**C(3), T(3)**	**C(3), T(3), H(3), O(3)**	**C(3), T(3), H(2), O(1)**
**FW**	**16–17.8.**	**FW**	**C(2), T(2), A(2)**	**C(2), T(2), A(2), H(2), O(2)**	

### Experimental set up

The experiment consisted of four treatments where copepods were fed 1) with water originating from a mesocosm with added nitrogen and phosphorus, either when food particles were dispersed (NP disp) or when they were aggregated (NP aggr), or copepods were fed 2) with water originating from a mesocosm with added nitrogen, phosphorus and silica using either dispersed (NPSi disp) or aggregated (NPSi aggr) food. All treatments were performed twice within a period of 5 d (Table 1). The measured variables were oxygen concentration to calculate respiration rates, fecal pellet accumulation to estimate egestion (fecal pellet production), and egg production (for the calanoids only). In addition to incubation in the different food regimes, egg and pellet production were measured in filtered sea water to determine the effect of feeding history on the measurements. Egg and pellet production of calanoids were determined by holding adult females in 0.5-l glass bottles at concentrations varying between 4 and 9 individuals bottle^-1^; pellet production of harpacticoids and *Oncaea* spp. (smaller species) were determined by using a mixture of late copepodite stages and females in 70-ml cell culture bottles at a concentrations of 4 to 10 individuals bottle^-1^. Late copepodite stages were included in the experiments with *Oncaea* sp. and harpacticoids due to the low amount of females in the net catches–therefore egg production measurements could not be conducted for these species. All incubations were performed in triplicate for each experiment, although the number of replicates was sometimes reduced because of the loss of copepods during the adaptation period (Table 1).

At the start of the incubations, experimental bottles were filled with the untreated mesocosm water for the dispersed treatments or with GF/F filtered seawater containing aggregates collected from the rolling tanks for aggregated treatments. Copepods were sorted out from the plankton sample using wide-mouth pipettes under a stereo microscope, and placed into experimental bottles to start the adaptation period. After 24-h of adaptation, the copepods were carefully filtered out using a submerged 50 μm sieve and transferred to new bottles containing fresh food (disp or aggr) where they were incubated for a 24 h experimental period. All bottles were incubated in a plankton wheel turning at the speed of 1 RPM in the dark and at a temperature of ca. 18°C. After approximately 24 h, copepods were carefully filtered onto 180 μm and eggs and pellets onto 15 μm sieves and flushed into Petri-dishes. The condition of copepods (dead or alive and active) was determined and the numbers of eggs and pellets produced were counted using a stereo microscope. The length and width of 30 pellets (if more than 30 present; as many as present if fewer) were measured using an ocular micrometer with a precision of 18 μm.

Actively swimming copepods were sorted out for respiration measurements immediately after the egg and pellet production incubations had been terminated. Respiration was measured for the groups of copepods originating from the same pellet production replicate if there were sufficient animals present at the end of incubation, but animals from the different replicates were pooled for the respiration measurements if not. Usually ≥ 80% of the individuals survived; the exception was *Acartia* spp. in NP aggregated treatment where the mortality was 56 ± 14%. For comparison, copepods in filtered seawater had an average mortality of 73 ± 13%.

Respiration was measured using an UNISENSE microrespirometer system, which consists of Clarke-type oxygen microsensors, an amplifier and a stirring system. The O_2_ concentration in sealed chambers was measured in 2 s intervals, and the data was logged using a MicOx software (www.unisense.com). The chambers were suspended in a temperature-controlled water bath. In present experiments, copepods were carefully pipetted to the 2-ml measurement chambers containing GF/F filtered seawater, the chamber was closed taking care that no copepods were lost, and placed into the water bath. After ca. 5 min, the O_2_ electrode was injected through the lid of the chamber and the measurements were started. Previous experiments with the microrespiration system showed that for *Calanus* spp. the smallest variation in measurements were obtained if the copepods were allowed to adapt for 5 min before starting the measurement and if the measurements were continued for at least 10 min. [34]. As the copepod species used here were considerably smaller, their measurements were continued for 30 min. The O_2_ concentration during this period never decreased by more than a few percentage points from the initial concentration. After a set of measurements, the contents of the chamber were emptied into a Petri dish, the condition of the copepods was checked and their prosome lengths were measured. Every set of respiration measurements included a 30-min. background measurement using only filtered seawater to determine microbial respiration rates and the oxygen consumption by the electrode.

### Experimental food concentration

Food concentration measured as chl-*a* was low during the first experiment in +NP mesocosm (0.22 μg l^-1^) and moderate during other experiments in both mesocosms (0.7–1.2 μg l^-1^; Table 2). All concentrations were likely to be below the saturation level for the calanoid copepods, assuming a carbon to chl-*a* ratio between 20 and 96 [35], and a saturating food concentration for feeding-current producing copepods at 300–500 μg C l^-1^ [36]. Food concentration measured as aggregates was approximately 100 small phytoplankton aggregates of < 1 mm in ESD (0.8 ± 0.6 mm) l^-1^. No visible aggregate formation was observed in dispersed treatments as a result of incubation in the plankton wheel.

**Table 2:** Concentration of chl-*a* (μg l^-1^) in mesocosm water at the time when the experiments were started.

**Experiment**	**Chl-*a***
**NP 1**	**0.22 ± 0.04**
**NP 2**	**0.98 ± 0.47**
**NPSi 1**	**1.2 ± 0.8**
**NPSi 2**	**0.7 ± 0.4**

We did not measure the decrease in food concentration during experiments, but based on the total food concentration in experimental bottles and maximum clearance rates of copepods in the size range of the individuals used in the present study, a food limitation was likely. The potential maximum clearance rates for *Centropages* sp., *Temora longicornis*, *Acartia* sp., harpacticoid spp. and *Oncaea* spp., are approximately 140, 120, 75, 40 and 7 ml ind.^-1^ d^-1^, respectively [8], suggesting that, in the absence of cell growth, most of the water in incubation bottles could have been cleared for food particles during the 24-h incubations. The relatively high physiological rates (see results), however, suggested that copepods in experiments did not suffer from severe food limitation, although the measured rates should be taken as conservative estimates. Also, in aggregated treatments, large numbers of aggregates were visible at the end of the experiments, similarly suggesting that the food source was not entirely depleted during the incubations.

### Analysis and statistics

Respiration rate was calculated from the rate of decrease of oxygen in time. The oxygen consumption of filtered seawater (background) was subtracted from the rate of respiration in chambers containing copepods to isolate copepod respiration rates. There were a few background measurements where the slope of the regression of oxygen against time was not significant, due to the low oxygen consumption of the filtered seawater. These measurements were excluded from further calculations. Also dropped were measurements with periods of clear electronic disturbances or of sudden jumps in measured oxygen concentrations caused by formation of air-bubbles.

The minimum carbon demand of copepods was calculated by summing C-specific rates of respiration, pellet production and egg production. Egg production was converted to carbon by using literature values for the egg sizes of *Acartia tonsa*, *Centropages typicus* and *Temora longicornis* [36, 37, 38]; female carbon content was calculated using measured prosome length (Table 3) and the length-weight regressions of Klein Breteler et al. [37] for *Temora longicornis* and *Centropages* sp. and Berggreen et al. [39] for *Acartia* spp. Pellet carbon was calculated from the measured volume (assuming a cylindrical shape; Table 3) and volume-specific carbon content from Ploug et al. [22] for *T*. *longicornis*, the average of Hansen et al. [40] and Butler and Dam [41] for *Acartia* sp., and the average of all three references for *Centropages* sp. Respiration was converted to carbon by using a respiratory quotient of 0.97 [25].

**Table 3:** Average body (harpacticoids) or prosome (calanoids and *Oncaea* spp.) length of females (μm) and average pellet volume (10^3^ μm^3^) in the dispersed and aggregated treatments of the two mesocosms (mean ± SD).

	Species	Female size (μm)	Pellet volume (10^3^ μm^3^)	
			Dispersed	Aggregated
**NP**	***Centropages* sp.**	**1014 ± 150**	255 ± 235 (114)*	**156 ± 89 (10)**
	***T*. *longicornis***	**760 ± 74**	271 ± 196 (219)*	**187 ± 187 (33)**
	***Acartia* sp.**	**850 ± 31**	146 ± 118 (46)*	**43 ± 42 (5)**
	**Harpacticoids**	**640 ± 38**	**57 ± 40 (40)**	**70 ± 62 (20)**
	***Oncaea* spp.**	**290 ± 4**	**55 ± 58 (14)**	**37 ± 32 (27)**
**NPSi**	***Centropages* sp.**	**1040 ± 70**	273 ± 194 (62)*	**101 ± 79 (14)**
	***T*. *longicornis***	**850 ± 33**	271 ± 177 (30)*	**177 ± 169 (25)**
	***Acartia* sp.**	**860 ± 61**	**123 ± 86 (49)**	**181 ± 306 (4)**
	**Harpacticoids**	**670 ± 37**	**97 ± 77 (15)**	**MD**
	***Oncaea* spp.**	**320 ± 20**	**84 ± 54 (16)**	**MD**

(*) Indicates pellet volumes which are significantly different between dispersed and aggregated treatments (1-way ANOVA on ranks; Dunn’s method; p < 0.05).

(MD) Missing data.

Egg and pellet production and respiration were tested first for differences between the two repeated experiments. As there were typically no significant differences between the two experiments (2-way ANOVA; p > 0.05; see Table 1 for dates), the data were pooled. Pellet and egg production were tested for differences between mesocosm media (NP vs. NPSi), experimental treatments (dispersed vs. aggregated) and copepod species, using a 3-way analysis of variance (ANOVA) followed by a Tukey HSD post hoc test. Respiration was tested for differences between mesocosm media, experimental treatments and species, using a 2-way ANOVA separately for mesocosms and experimental treatments (Table 4), due to missing data for *Acartia* sp. in one of the treatments (Table 1). Both individual and weight-specific rates (in carbon) were tested, but since there were no differences in main trends, only ANOVA results for absolute rates are given. To investigate if the respiration and egg production were related to feeding (pellet production), a Pearson correlation analysis was conducted, separately for each species. All data was tested for normality and equal variance, and if the conditions were not met, log or square-root transformed. For pellet volume, transforming the data did not solve the problems with the normality and ANOVA on ranks and Dunn’s post hoc test were used, separately for each species (Table 4). All tests were conducted using a Sigmastat 3.5 statistical package.

**Table 4:** Parameters from 1, 2 and 3-way analysis of variance (ANOVA) or Kruskal-Wallis ANOVA on ranks testing the differences in pellet production, pellet volume, respiration and egg production between the mesocosms media (MK), experimental treatments (Treat) and copepod species.

Variable	Type	MK (NP vs. NPSi)	Treat (Aggr. vs. Disp.)	Species	Interactions
**Pellet production**	**3-way**	**F**_**1,84**_ = 19.6***	**F**_**1,84**_ = 8.1**	**F**_**4,84**_ = 5.6***	Treat x Species***
**Pellet volume-C**	**1-way on ranks**	**H**_**3**_ = 16.9***			
**Pellet volume-T**	**1-way on ranks**	**H**_**3**_ = 17.0***			
**Pellet volume-A**	**1-way on ranks**	**H**_**3**_ = 9.6*			
**Pellet volume-H**	**1-way on ranks**	**Ns**			
**Pellet volume-O**	**1-way on ranks**	**H**_**2**_ = 13.2***			
**Respiration-MK**	**2-way**	**F**_**1,26**_ = 27.1***		**F**_**3,26**_ = 7.0**	**Ns**
**Respiration-Treat**	**2-way**		**F**_**1,24**_ = 20.1***	**Ns**	Treat x Species**
**Egg production**	**3-way**	**Ns**	**F**_**1,56**_ = 48.7**	**F**_**2,56**_ = 5.0*	**Ns**

(*) Significant at the p < 0.05 level

(**) significant at the p < 0.01 level

(***) significant at the p < 0.001 level. (ns) Not statistically significant (p > 0.05).

Other abbreviations as in Table 1.

## Results

### Differences between dispersed and aggregated treatments

#### Pellet production

**Fig 1:**
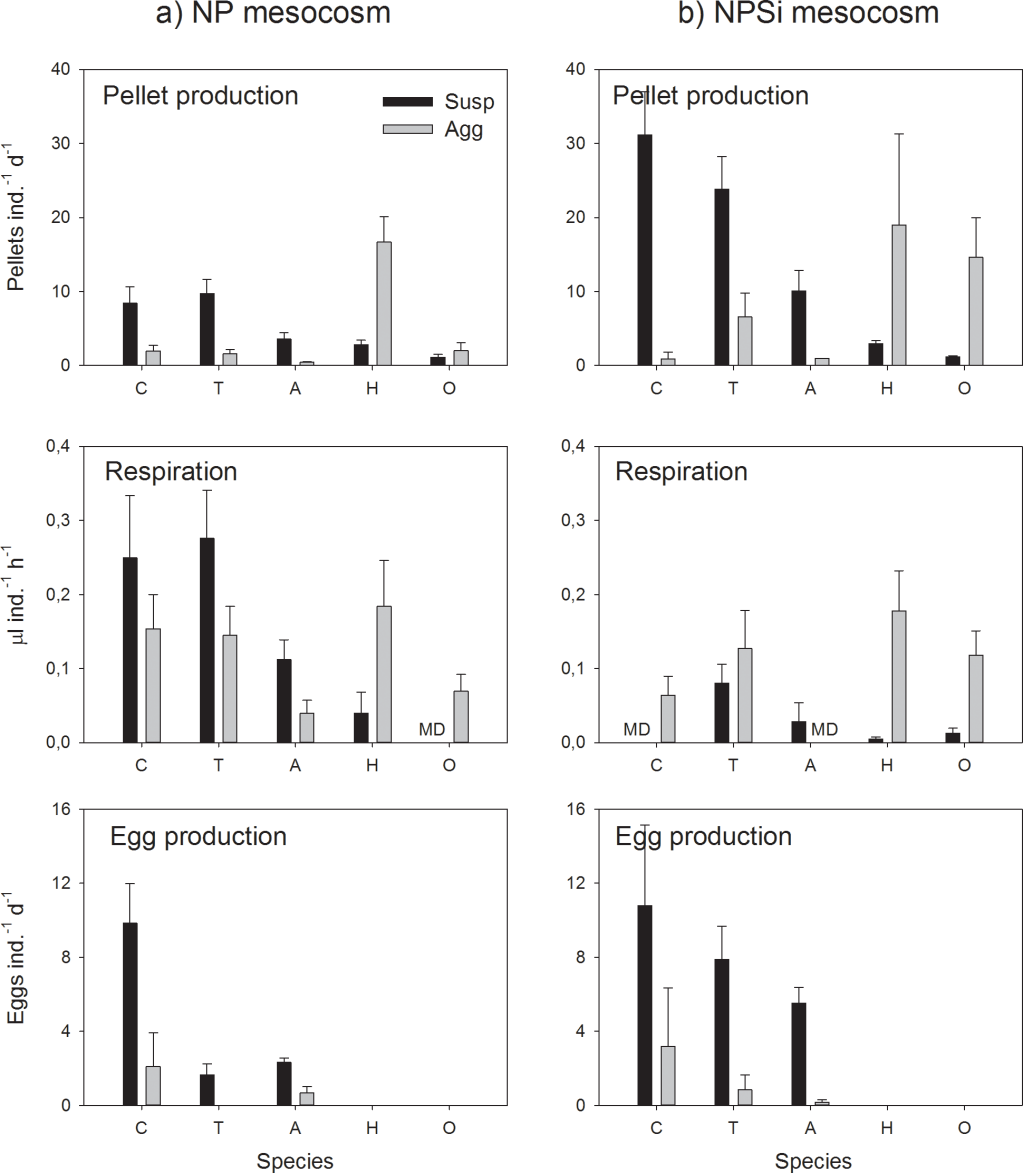
Physiological rates of the copepods. Pellet production (pellets ind.^-1^ d^-1^), respiration (μl ind.^-1^ h^-1^) and egg production (eggs f^-1^ d^-1^) of *Centropages* sp. (C), *Temora longicornis* (T), *Acartia* sp. (A), harpacticoid sp. (H) and *Oncaea* spp. (O) incubated in the water originating from a) mesocosm without silica addition (NP) and b) mesocosm with added silica (NPSi; mean ± SE). Dark columns indicate treatments with dispersed food, light grey columns treatments with aggregated food. (MD) Missing data, (blank) no experiment.

Pellet production of *Centropages* sp., *Temora longicornis* and *Acartia* sp. was 4–10 times greater when feeding on dispersed food rather than on aggregates. In contrast, pellet production of *Oncaea* spp. and harpacticoids was 2–12 higher when feeding on aggregated food than on dispersed food. Thus, calanoids fed overwhelming on dispersed food, *Oncaea* and harpacticoids fed predominantly on aggregates (Fig 1), which resulted in a significant interaction between the treatment and copepod species (Table 4). In fact, the pellet production of all species (except for *Oncaea* spp. feeding on NP food) was significantly different between dispersed and aggregated food (Tukey HSD; p < 0.05). Pellet production of calanoids increased with increasing food concentration in dispersed treatments, while pellet production in aggregated treatments was always equally low, and close to the pellet production of starved copepods (< 0.5 pellets ind.^-1^ d^-1^, irrespective of species; S1 Fig).

Average volume of individual pellets from calanoid copepods was significantly greater for copepods feeding on dispersed food than that of animals feeding on aggregates, pellet volume of harpacticoids and *Oncaea* spp. was variable but not significantly different between treatments (Tables 3 and 4). The pellet size thus did not change the trend between dispersed vs. aggregated treatments, but weight-specific pellet production of calanoids was typically highest with dispersed food (0.12–0.39 μg C (μg C)^-1^ d^-1^) and lowest with aggregated food (≤ 0.01 μg C (μg C)^-1^ d^-1^). Harpacticoids had a high weight-specific pellet production in aggregated food (0.16–0.20 μg C (μg C)^-1^ d^-1^) and low one in dispersed food (0.006–0.03 μg C (μg C)^-1^ d^-1^). Weight-specific pellet production of *Oncaea* spp. was always relatively low, mainly due to the small size of their pellets (Tables 3 and 5).

**Table 5:** Average weight-specific respiration, pellet production and egg production (μg C (μg C)^-1^ d^-1^) in the dispersed and aggregated treatments of the two mesocosm (mean ± SD). (Blank) No experiment, (MD) missing data.

	Copepod	Respiration		Pellet production		Egg production	
		Dispersed	Aggregated	Dispersed	Aggregated	Dispersed	Aggregated
**NP**	***Centropages* sp.**	**0.62 ± 0.53**	**0.27 ± 0.18**	**0.15 ± 0.12**	**0.01 ± 0.01**	**0.06 ± 0.03**	**0.01 ± 0.02**
	***T*. *longicornis***	**0.70 ± 0.37**	**0.31 ± 0.25**	**0.11 ± 0.06**	**0.01 ± 0.01**	**0.01 ± 0.01**	**0 ± 0**
	***Acartia* sp.**	**0.36 ± 0.16**	**0.12 ± 0.07**	**0.07 ± 0.05**	**0.002 ± 0.001**	**0.03 ± 0.01**	**0.01 ± 0.001**
	**Harpacticoids**	**0.24 ± 0.37**	**0.84 ± 0.43**	**0.006 ± 0.004**	**0.16 ± 0.06**		
	***Oncaea* spp.**	**MD**	**2.40 ± 1.86**	**0.06 ± 0.04**	**0.006 ± 0.004**		
**NPSi**	***Centropages* sp.**	**MD**	**0.10 ± 0.07**	**0.36 ± 0.20**	**0.005 ± 0.01**	**0.05 ± 0.04**	**0.01 ± 0.03**
	***T*. *longicornis***	**0.14 ± 0.10**	**0.24 ± 0.22**	**0.19 ± 0.09**	**0.04 ± 0.05**	**0.04 ± 0.02**	**0.004 ± 0.01**
	***Acartia* sp.**	**0.10 ± 0.13**	**MD**	**0.12 ± 0.07**	**0.01 ± 0**	**0.07 ± 0.03**	**0.001 ± 0.002**
	**Harpacticoids**	**0.02 ± 0.02**	**0.89 ± 0.53**	**0.03 ± 0.01**	**0.20 ± 0.23**		
	***Oncaea* spp.**	**0.32 ± 0.31**	**2.87 ± 1.52**	**0.007 ± 0.001**	**0.03 ± 0.02**		

#### Respiration

Despite the gaps in the data, the trend in respiration was similar than in pellet production. Respiration of calanoids was typically higher in dispersed than aggregated food, and respiration of harpacticoids was significantly higher in aggregated than dispersed food (Fig 1). Treatment (aggregated vs. dispersed) thus induced significant differences in respiration rates (Table 4), but with species-specific responses (Fig 1).

Weight-specific respiration of calanoids (in carbon) was typically 0.12–0.36 μg C (μg C)^-1^ d^-1^, with the exception of a high and variable respiration of *Centropages* sp. and *Temora longicornis* in (NP) dispersed treatment (Table 5). Weight-specific respiration of harpacticoids was relatively low in dispersed treatments (0.02–0.2 μg C (μg C)^-1^ d^-1^) and high (0.8–0.9 μg C (μg C)^-1^ d^-1^) in aggregated treatments. *Oncaea* spp. in aggregated treatments had an unrealistically high weight-specific respiration rate, several times higher than their body carbon consumed in one day (2.4–2.9 μg C (μg C)^-1^ d^-1^), a result which could not be explained with the variability in the data. In general, the respiration of *Oncaea* spp. had a different weight-specific rate than did the respiration of calanoids.

#### Egg production

Egg production of calanoids was always higher in dispersed treatment than in aggregated treatment and higher for *Centropages* sp. than for *Temora longicornis* or *Acartia* sp. (Fig 1, Table 4; Holm Sidak method; p < 0.05). The egg production in dispersed treatment varied from 2 to 11 eggs f^-1^ d^-1^ (0.028–0.05 μg C (μg C)^-1^ d^-1^), while egg production in aggregated treatments was never higher than a few eggs f^-1^ d^-1^ for any of the species (Fig 1). No eggs were produced in filtered sea water.

#### Carbon demand

**Fig 2:**
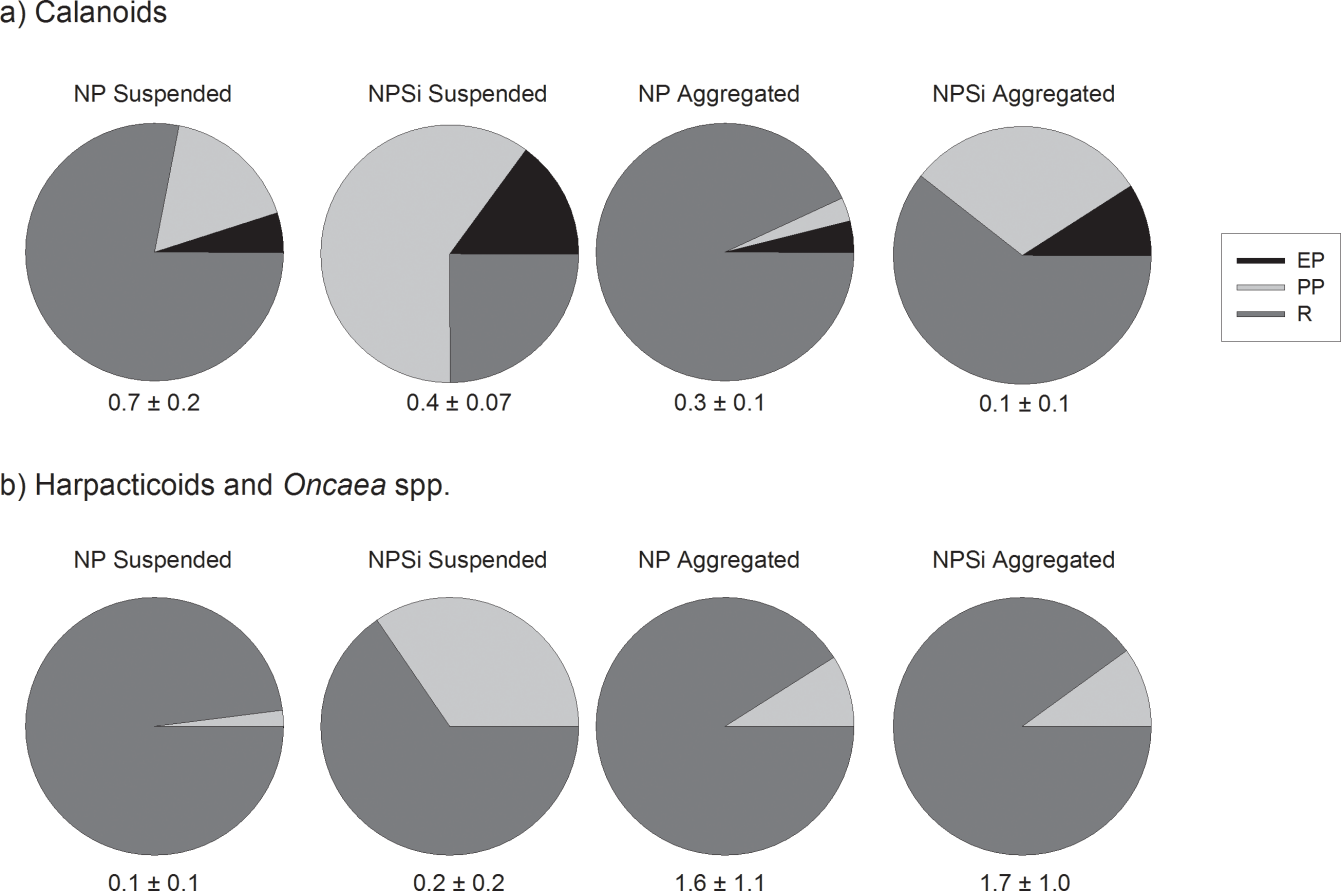
Division of individual carbon budgets for suspension and aggregate feeders. Contribution of egg production (EP), pellet production (PP) and respiration (R) to the weight-specific carbon consumption (μg C (μg C)^-1^ d^-1^) of a) calanoids and b) harpacticoids and *Oncaea* spp. in the dispersed and in the aggregated food. The egg production, pellet production and respiration represent the average of all calanoid species (a) or the average of harpacticoids and *Oncaea* spp. (b) presented in Table 5. Note that the carbon budget for harpacticoids and *Oncaea* in dispersed treatments is based on fewer measurements than the other treatments (cf. Table 1). The total average carbon demand of all species is indicated in the figure (μg C (μg C)^-1^ d^-1^; mean ± SD).

The total carbon demand of calanoid copepods varied between 0.1 and 0.7 μg C (μg C)^-1^ d^-1^, being 2–3 times higher in dispersed than in aggregated treatments (Fig 2A), while the total carbon demand of harpacticoids and *Oncaea* was >7 times higher in aggregated than in dispersed treatments (Fig 2B). The carbon demand of copepods experiencing non- preferred food environment was similar for calanoids, harpacticoids and *Oncaea* spp. (around 10% body weight^-1^), while the carbon demand of harpacticoids and *Oncaea* spp. in their preferred food environment was 2–3 times higher than that of calanoids (Fig 2), possibly reflecting the patchy and unpredictable food availability of particle-feeding copepods.

### Differences between silicon-rich and silicon-poor food

The effect of treatment (dispersed vs. aggregated) typically overruled the effect of media (Si-rich vs. Si-poor) in all experiments. However, silicon-rich food source typically induced a higher pellet production than the silicon-poor food, with a similar effect for all species (Fig 1; Table 4) although there was no effect of media on pellet size (Table 3). Media had a significant species-specific effect on respiration (Fig 1). In general, calanoids had a higher respiration rate in NP media than in NPSi media, while there was no significant difference between media in *Oncaea* sp. and harpacticoids (Tukey HSD; p > 0.05).

Silicon-rich and silicon-poor food induced similar egg production of *Centropages* sp. which produced on average 11 eggs f^-1^ d^-1^ in both NP and NPSi media. This corresponded to a weight-specific egg production of ca. 0.05 μg C (μg C)^-1^ d^-1^ (Table 5). Egg production of both *T*. *longicornis* and *Acartia* sp. was higher in NPSi media than NP media; in the case of *T*. *longicornis* the higher egg production was also related to the higher pellet production in NPSi media (Pearson; 0.946; p < 0.0001). Egg production of *T*. *longicornis* in NP and NPSi media was 1.7 and 7.9 eggs f^-1^ d^-1^ (0.01 and 0.04 μg C (μg C)^-1^ d^-1^), respectively. *Acartia* sp. egg production was 2.3 eggs f^-1^ d^-1^ (0.028 μg C (μg C)^-1^ d^-1^) in NP and 5.5 eggs f^-1^ d^-1^ (0.07 μg C (μg C)^-1^ d^-1^) in NPSi media, and did not correlate with the pellet production.

Silicon-rich and silicon-poor media induced different partitioning of carbon both in calanoids and in harpacticoids and *Oncaea* sp. (Fig 2). While nearly all of the carbon demand in NP media was due to respiration, pellet production accounted for 30–60% of the carbon demand of both types of copepods in the dispersed NPSi treatment, probably due to a high pellet production in a diatom-dominated food environment (see [21, 38]).

## Discussion

We suggest that copepods divide into those primarily suspension feeders and those primarily aggregate feeders. The suspension feeders consist mainly of calanoids, and consume sinking aggregates only to a limited extent. The aggregate feeders include species which are frequently observed on marine snow particles such as *Microsetella norvegica*, other harpacticoids and *Oncaea* spp. [14], and have a limited ability to feed on small dispersed particles [12]. The three calanoid copepods used in the present study are typical neritic species ranging in length from 700 to 1000 μm. Although substantially smaller than many oceanic calanoids, their feeding mode [42] and vertical migration behavior [43, 44] should be representative of calanoids (such as *Calanus* spp.) using feeding-current and migrating vertically. Similarly, *Oncaea* spp. and harpacticoids have a feeding mode and behavior representing particle-colonising species in general [11]; in the open ocean these copepods would mainly consist of different *Oncaea* species and of a pelagic harpacticoid *Microsetella norvegica* [45].

### Physiological rates compared to other studies

Copepods in these experiments fed actively when the food was available in a form they could utilize, but were close to starvation when the food was not available in their preferred form. Pellet production rates of calanoids in dispersed treatments of +NP and +NPSi mesocosms corresponded to rates typically measured for diverse calanoid species feeding predominantly on flagellates or diatoms at non-saturated concentrations [21, 22, 23], while calanoid pellet production in aggregated treatments was close to values for starved copepods, similar to the observations of Bochdansky and Herndl with *Acartia* sp. [46]. Only *Temora* sp. appeared to be able to extract food in aggregated +NPSi treatment; its pellet production of ca. 5 pellets ind.^-1^ d^-1^ was very similar to the rates measured for *Temora longicornis* feeding on appendicularian houses [18].

The pellet production of harpacticoids and *Oncaea* spp. in aggregated treatments indicated moderate to high feeding rates; for instance *Tisbe carolinensis* can produce > 30 pellets d^-1^ when feeding on diatoms, while pellet production rates are 10-time lower on flagellate diets [47]. In addition, pellet production rates were generally higher in the NPSi mesocosm, which could either reflect the higher food concentration during the first experiment (see Table 2), or the dominance of diatoms inducing lower assimilation (in the present study demonstrated as a high pellet production compared to respiration and egg production) in NPSi mesocosm. The high proportion of pellet production from the total carbon consumption with diatom diets is a feature typically observed for diverse calanoid copepods, both for small neritic species [21] and the large oceanic ones [48].

It is possible that the food concentration in aggregated treatments was lower than in dispersed treatments confounding the comparison between dispersed and aggregated feeding. While it was clear that *Oncaea* spp. and harpacticoids were able to extract food from aggregated treatments irrespective of the food concentration, the feeding of calanoids could have been inhibited by a low food concentration rather than the form of food, particularly if the species were predominantly feeding by producing a feeding current with no feeding below a threshold food concentration. This type of functional response has been observed for *Calanus pacificus* feeding on marine snow particles, resulting in substantial feeding rates only at marine snow concentrations above approximately 500 μg C l^-1^ [17].

The calanoids in our experiment should be able to both feed by producing a feeding current and by using ambush feeding [8, 49, 50], which should have allowed them to encounter and consume marine snow particles at the experimental food concentration of 100 aggregates l^-1^ [18]. Unfortunately we did not measure the food concentration in chl-*a* or carbon in aggregated treatments. The differences in chl-*a* concentration and phytoplankton community composition between mesocosms should nevertheless have resulted in a range of food concentration in aggregate treatments, for instance due to more aggregation and larger and denser aggregates in diatom-dominated mesocosms [51]. However, the pellet production of calanoid copepods remained close to starvation rates in all treatments (S1 Fig), suggesting that feeding on aggregates was inefficient at concentrations around 100 aggregates l^-1^, although feeding on aggregates by calanoid copepods might take place at higher aggregate concentrations.

Respiration rates generally confirmed the trend observed in feeding (pellet production) rates with lower rates when copepods were starving and higher rates when they were actively feeding, as would be expected due to specific dynamic action [27, 36]. Respiration rate of *Acartia* spp. in aggregated treatment at 0.04 μl ind.^-1^ h^-1^ was generally similar to previous estimates for the basal metabolism of the same species: 0.05 μl ind.^-1^ h^-1^ at ‘routine metabolism’ if estimated based on body size and temperature [25, 26], or 0.01 or 0.04 μl ind.^-1^ h^-1^ for starved copepods [36, 52]. Respiration rate in dispersed treatment (NP mesocosm only) was circa double of the respiration in aggregated treatment, similar to Kiørboe et al. [36] and Thor [27, 28]. Similar 2-fold difference in respiration rates between aggregated and dispersed treatments was observed for *Centropages* sp. and *Temora* sp. in NP mesocosm, although the non-feeding respiration at 0.15 μl ind.^-1^ h^-1^ was higher than what would be estimated for routine metabolism based on body size and temperature (0.08–0.09 μl ind.^-1^ h^-1^ according to Ikeda) [25, 26].

*Oncaea* spp. have typically lower weight-specific respiration rates than calanoids [29, 52], ranging from < 0.001 μl ind.^-1^ h^-1^ measured for *Triconia borealis* at 3°C to 0.03 μl ind.^-1^ h^-1^ measured for *Oncaea* spp. at 20°C [52]. The respiration rates of harpacticoids appear to have a similar large range, for instance Herman and Heip [53] measured respiration rates ranging from 0.003 to 0.02 μl ind.^-1^ h^-1^ for small meiobenthic harpacticoids. The respiration rates measured for harpacticoids and *Oncaea* spp. in dispersed treatments of the mesocosm were similar to these observations, ranging from 0.005 to 0.04 μl ind.^-1^ h^-1^. However, the respiration rates in aggregated treatments were extremely high, up to 0.2 μl ind.^-1^ h^-1^, which in the case of *Oncaea* spp. would correspond to a minimum daily carbon concentration of several times the body size. Although part of the unrealistically high weight-specific rates could be due to uncertainties of estimating the carbon weight based on length, the increase in respiration rate due to feeding in NPSi mesocosm was > 10 fold. Koski et al. [12] estimated short-term feeding rates of starved harpacticoids to be > 10 times higher than the daily average, suggesting that copepods could fill their guts during short visits to marine snow aggregates. If the feeding activity of harpacticoids in the aggregated treatments was similar to these short-term rates, also a high specific dynamic action could be expected. The variable feeding conditions might be more important in influencing the respiration measurements than, for instance, hydrostatic pressure (the water layer from which the copepods originate; [54]), and large fluctuations in food supply could cause variation in allometric relationships used to estimate the carbon demand of the mid-water communities, particularly if the community is dominated by particle-colonizing species.

Egg production of calanoids was low if compared to maximum rates measured from e.g., North Sea [55]. This could be due to the late season (August) as all calanoids used in the study typically have their peak egg production during the spring–early summer [55]. Egg production of *Temora longicornis* was, however, also related to pellet production, with more eggs produced with a higher feeding rate in the mesocosms dominated by diatoms, which could indicate that the egg production in NP mesocosms was limited by qualitative or quantitative food resources. Egg production of calanoids during their peak reproduction would typically be around 30% of the ingested carbon, but that could vary largely depending on season, food quality and quantity [56]. The present egg production of 5–15% of the minimum carbon consumption could thus represent any copepod community outside their peak reproductive season.

### Potential implications for vertical flux

The sum of respiration, pellet production and egg production in our experiments resulted in a daily carbon demand of starved copepods of around 10% body weight^-1^, while the carbon demand of feeding calanoids and harpacticoids / *Oncaea* spp. was 2–3 and > 7 times higher, respectively. This indicates that the species composition combined with the food source could have large influence both on the short-term carbon demand of a copepod community and on the quality and quantity of sinking flux. Our results suggest that a community dominated by small particle colonisers would primarily be feeding on sinking aggregates, while a calanoid-dominated community could mainly feed on dispersed phyto- and microzooplankton and potentially repackage these into large fast sinking fecal pellets, particularly when the food is dominated by diatoms [23, 41]. While our study focused on the differences between feeding on dispersed food and feeding on aggregated food, it should be remembered that aggregates can have a different size, consistency and origin [57], which might result in different feeding rates of zooplankton. Although not much is known about the effects of aggregate composition on zooplankton feeding, our previous experiments suggest differences in encounter rate and handling time of copepods depending on the aggregate type [Koski et al., unpubl.], which, together with the aggregate size, could influence the species composition and feeding rates of the particle-colonizing community.

A resident mid-water copepod community can consist of both calanoid copepods and particle-feeders, with the proportional importance of different groups changing with e.g., depth [58, 59]. In many Arctic areas the copepod community is dominated by small species, particularly later in the season [60], and a shift towards smaller species is suggested to occur with warming sea surface temperatures [61]. Fluctuations in the zooplankton community structure as shown for instance in the North Atlantic [62, 63], could thus have a large effect on the flux attenuation by zooplankton. If we calculate the minimum carbon demand of a copepod community using a composition of the particulate organic matter [64] and biomasses of calanoids (represented by *Calanus* spp.) and particle colonisers (sum of *Microsetella norvegica* and *Oncaea* spp.) from the North Atlantic [Koski et al., unpubl.], together with food type -specific and copepod species—specific physiological rates from the present study, the resulting estimates are many-fold different from the estimates obtained based on available models relating respiration to body size and temperature [25] (Table 6). For calanoids, the carbon demand based on the measured physiological rates would be approximately five times higher than a rate obtained by using a model, while for particle-coloniser, the carbon demand would be overestimated during a period of low aggregate abundance, but greatly underestimated when aggregates are abundant (Table 6), for instance during a post spring bloom period. It thus appears that the copepod species and the availability of the food in the form which they can utilize can have many-fold effect on the flux attenuation by copepod community, and dividing the species into suspension vs. aggregate feeders could be a reasonable alternative for a size-class based division when modeling the vertical flux. In any case, understanding the factors regulating the zooplankton community structure as well as its effect on the biological carbon pump should have a high priority in future zooplankton studies.

**Table 6:** Estimation of the total carbon demand (mg C m^-2^ d^-1^) of the copepod community in the North Atlantic (Iceland Basin) in April 2012 as well as the percentage contribution by particle-colonizing copepods (in parenthesis), using experimental rates of carbon demand obtained in the present study (exp) compared to a modeled rate based on the average body size and temperature according to [25] (mod; μg C (μg C)^-1^ d^-1^). During the first sampling date most of the phytoplankton was present as dispersed particles, while a few weeks later, approximately one third of the POC consisted of aggregates [64]. We thus assumed that there was a sufficient availability of dispersed food for calanoid copepods at both sampling dates, while aggregated food was only available on the later date. The respiration rates measured in the present study were corrected for temperature using a Q_10_ of 2.0 for calanoids [25] and Q_10_ of 3.1 for particle-colonising copepods [65]. Total particulate organic carbon (POC; mg C m^-2^) and the ratio of dispersed vs. aggregated particles (in carbon) are calculated as averages for 10^th^ April and 21^st^ April by using the sum of small and large sinking particles to represent aggregated particles (Table 1 in [64]). Copepod biomass (mg m^-2^) is based on sampling with Multinet Midi (Hydrobios) using 50 μm mesh size on 10^th^ and 29^th^ April [Koski et al., unpubl].

	Mid April	End April	References
**Total POC**	**95.1**	**96.2**	**[64]**
**Dispersed: Aggregated POC**	**21.7 ± 12**	**2.6 ± 1.2**	**[64]**
***Calanus* spp. BM**	**1340**	**1800**	**Koski et al. unpubl.**
**Particle-colonising BM**	**113**	**1030**	**Koski et al. unpubl.**
***Calanus* spp. carbon demand exp**	**0.27 ± 0.13**	**0.27 ± 0.13**	**This study**
**Particle-colonising carbon demand exp**	**0.06 ± 0.02**	**0.5 ± 0.1**	**This study**
***Calanus* spp. carbon demand mod**	**0.05**	**0.06**	**[25]**
**Particle-colonising carbon demand mod**	**0.15**	**0.16**	**[25]**
**Total carbon demand exp**	**369 (2)**	**1001 (51)**	
**Total carbon demand mod**	**82 (20)**	**263 (61)**	

## Supporting information

S1 Fig The effect of food concentration on pellet production rates. Pellet production (pellets ind.^-1^ d^-1^) of *Centropages* sp., *Temora longicornis* and *Acartia* sp. as a function of Chl-*a* concentration (μg l^-1^) in mesocosms media in a) aggregated and b) dispersed treatments (mean ± SD). (Closed circles) *Centropages* sp., (open circles) *T*. *longicornis*, (grey circles) *Acartia* sp.
(JPG)

## References

[1] Martin JH, Knauer GA, Karl DM, Broenkow WW. VERTEX: Carbon cycling in the northeast Pacific. Deep-Sea Res. 1987; 34: 267–285.

[2] Wassmann P. Retention versus export food chains: processes controlling sinking loss from marine pelagic systems. Hydrobiol. 1998; 363: 29–57.

[3] Buesseler KO, Lamborg CH, Boyd PW, Lam PJ, Trull TW, Bidigare RR, et al Revisiting carbon flux through the ocean’s twilight zone. Science 2007; 316: 567–569. doi: 10.1126/science.1137959 17463282 10.1126/science.1137959

[4] Steinberg D, Van Mooy BAS, Buesseler KO, Boyd PW, Kobari T, Karl DM. Bacterial vs. zooplankton control of sinking particle flux in the ocean’s twilight zone. Limnol. Oceanogr. 2008; 53: 1327–1338.

[5] Iversen MH, Ploug H. Ballast minerals and the sinking carbon flux in the ocean: carbon-specific respiration rates and sinking velocity of marine snow aggregates. Biogeosci. 2010; 7: 2613–2624.

[6] Stukel MR, Landry MR, Benitez-Nelson CR, Goerickea R. Trophic cycling and carbon export relationships in the California Current Ecosystem. Limnol. Oceanogr. 2011; 56: 1866–1878.

[7] Wilson SE, Ruhl HA, Smith KL Jr. Zooplankton fecal pellet flux in the abyssal northeast Pacific: A 15 year time-series study. Limnol. Oceanogr. 2013; 58: 881–892.

[8] Kiørboe T. How zooplankton feed: Mechanisms, traits and tradeoffs. Biol. Rev. 2011; 86: 311–340. doi: 10.1111/j.1469-185X.2010.00148.x 20682007 10.1111/j.1469-185X.2010.00148.x

[9] Hansen B, Bjørnsen PK, Hansen PJ. The size ratio between planktonic predators and their prey. Limnol. Oceangr. 1993; 39: 395–403.

[10] Svendsen C, Kiørboe T. Remote prey detection in *Oithona similis*: hydromechanical versus chemical cues. J. Plankton Res. 2000; 22: 1155–1166.

[11] Jackson G. Flux feeding as a mechanism for zooplankton grazing and its implications for vertical particle flux. 1993; 38: 1328–1331.

[12] Koski M, Kiørboe T, Takahashi K. Benthic life in the pelagial: aggregate encounter and degradation rates by pelagic harpacticoid copepods. Limnol. Oceanogr. 2005; 50: 1254–1263.

[13] Steinberg DK, Silver MW, Pilskaln CH, Coale SL, Paduan JB. Midwater zooplankton communities on pelagic detritus (giant larvacean houses) in Monterey Bay, California, USA. Limnol. Oceanogr. 1994; 39: 1606–1620.

[14] Green EP, Dagg MJ. Mesozooplankton associations with medium to large marine snow aggregates in the northern Gulf of Mexico. J. Plankton Res. 1997; 19: 435–447.

[15] Alldredge AL. Abandoned larvacean houses: a unique food source in the pelagic environment. Science 1972; 177: 885–887. doi: 10.1126/science.177.4052.885 17780989 10.1126/science.177.4052.885

[16] Kiørboe T. Colonisation of marine snow aggregates by invertebrate zooplankton: abundance, scaling, and possible role. Limnol. Oceanogr. 2000; 45: 479–484.

[17] Dilling L, Wilson J, Steinberg D, Alldredge A. Feeding by the euphausiid *Euphasia pacifica* and the copepod *Calanus pacificus* on marine snow. Mar. Ecol. Prog. Ser. 1998; 170: 189–201.

[18] Lombard F, Koski M, Kiørboe T. Copepods use chemical trails to find sinking marine snow aggregates. Limnol. Oceanogr. 2013; 58: 185–192.

[19] Dagg M. Sinking particles as a possible source of nutrition for the large calanoic copepod *Neocalanus cristatus* in the subarctic Pacific Ocean. Deep-Sea Res. I. 1993; 40: 1431–1445.

[20] Wilson SE, Steinberg DK. Autotrophic picoplankton in mesozooplankton guts: evidence of aggregate feeding in the mesopelagic zone and export of small phytoplankton. Mar. Ecol. Prog. Ser. 2010; 412: 11–27.

[21] Besiktepe S, Dam HG. Coupling of ingestion and defecation as a function of diet in the calanoid copepod *Acartia tonsa*. Mar. Ecol. Prog. Ser. 2002; 229: 151–164.

[22] Ploug H, Iversen MH, Koski M, Buitenhuis ET. Production, oxygen respiration rates, and sinking velocity of copepod fecal pellets: direct measurements of ballasting by opal and calcite. Limnol. Oceanogr. 2008; 53: 469–476.

[23] Feinberg LR, Dam HG. Effects of diet on dimensions, density and sinking rates of fecal pellets of the copepod Acartia tonsa. 1998; Mar. Ecol. Prog. Ser. 175: 87–96.

[24] Møller EF, Andersen Borg CA, Jonasdottir SH, Satapoomin S, Jaspers C, Nielsen TG. Production and fate of copepod fecal pellets across the Southern Indian Ocean. Mar. Biol. 2011; 158: 677–688.

[25] Ikeda T, Kanno Y, Ozaki K, Shinada A. Metabolic rates of epipelagic marine copepods as a function of body mass and temperature. Mar. Biol. 2001; 139: 587–596.

[26] Ikeda T, Sano F, Yamaguchi A. Respiration in marine pelagic copepods: a global-bathymetric model. Mar. Ecol. Prog. Ser. 2007; 339: 215–219.

[27] Thor P. Specific dynamic action and carbon incorporation in *Calanus finmarchicus* copepodites and females J. Exp. Mar. Biol. Ecol. 2002; 272: 159–169.

[28] Thor P. Elevated respiration rates of the neritic copepod *Acartia tonsa* during recovery from starvation. J. Exp. Mar. Biol. Ecol. 2003; 283:133–143.

[29] Nishibe Y, Ikeda T. Metabolism and elemental composition of four oncaeid copepods in the western subarctic Pacific. Mar. Biol. 2008; 153: 397–404.

[30] Hays GC. Ontogenetic and seasonal variation in the diel vertical migration of the copepods *Metridia lucens* and *Metridia longa*. Limnol. Oceanogr. 1995; 40: 1461–1465.

[31] Burd AB, Hansell DA, Steinberg DK, Anderson TR, Arístegui J, Baltar F, et al Assessing the apparent imbalance between geochemical and biochemical indicators of meso- and bathypelagic biological activity: What the @$]! Is wrong with present calculations of carbon budgets? Deep-Sea Res. II. 2010; 57: 1557–1571.

[32] Giering SLC, Sanders R, Lampitt RS, Anderson TR, Tamburini C, Boutrif M, et al Reconciliation of the carbon budget in the ocean’s twilight zone. Nature 2014; 507: 480–483. doi: 10.1038/nature13123 24670767 10.1038/nature13123

[33] Shanks AL, Edmondson EW. Laboratory-made artificial marine snow: a biological model of the real thing. Mar. Biol. 1989; 101: 463–70.

[34] Pankoke LM. Diurnal and spatial changes in the respiration rate of Calanus spp. in the North Atlantic. M.Sc. Thesis, Technical University of Denmark. 2012.

[35] Jakobsen HH, Markager S. Carbon-to-chlorophyll ratio for phytoplankton in temperate coastal waters: Seasonal patterns and relationship to nutrients. 2016; Limnol. Oceanogr. 61: 1853–1868.

[36] Kiørboe T, Møhlenberg F, Hamburger K. Bioenergetics of the planktonic copepod *Acartia tonsa*: relation between feeding, egg production and respiration, and the composition of specific dynamic action. Mar. Ecol. Prog. Ser. 1985; 26: 85–95.

[37] Klein Breteler WCM, Fransz HG, Gonzales SR. Growth and development of four calanoid copepod species under experimental and natural conditions. Neth. J. Sea Res. 1982; 16: 195–207.

[38] Dutz J, Koski M, Jónasdóttir SH. Copepod reproduction is unaffected by diatom aldehydes or lipid composition. Limnol. Oceanogr. 2008; 53: 225–235.

[39] Berggreen U, Hansen B, Kiørboe T. Food size spectra ingestion and growth of the copepod *Acartia tonsa* during development: implications for determination of copepod production. Mar. Biol. 1988; 99: 341–352.

[40] Hansen B, Fotel FL, Jensen NJ, Madsen SD. Bacteria associated with a marine planktonic copepod in culture. II. Degradation of fecal pellets produced on a diatom, a nanoflagellate or a dinoflagellate diet. J. Plankton Res. 1996; 18: 275–288.

[41] Butler M, Dam HG. Production rates and characteristics of fecal pellets of the copepod *Acartia tonsa* under simulated phytoplankton bloom conditions: implications for vertical fluxes. Mar. Ecol. Prog. Ser. 1994; 114: 81–91.

[42] Paffenhöfer GA, Strickler JR, Alcaraz M. Suspension-feeding by herbivorous calanoid copepods: A cinematographic study. Mar. Biol. 1982; 67: 193–199.

[43] Pagano M, Gaudy R, Thibault D, Lochet F. Vertical Migrations and Feeding Rhythms of Mesozooplanktonic Organisms in the Rhône River Plume Area (North-west Mediterranean Sea). Est. Coast. Shelf Sci. 1993; 37: 251–269.

[44] Lo W-T, Shih C-T, Hwang J-S. Diel vertical migration of the planktonic copepods at an upwelling station north of Taiwan, western North Pacific. J. Plankton Res. 2004; 26: 89–97.

[45] Turner J. The Importance of Small Planktonic Copepods and Their Roles in Pelagic Marine Food Webs. Zool. Stud. 2004; 43: 255–266.

[46] Bochdansky AB, Herndl GJ. Ecology of amorphous aggregations (marine snow) in the Northern Adriatic Sea: Zooplankton interactions with marine snow. 1992; Mar. Ecol. Prog. Ser. 87: 135–146.

[47] Lee Y, Zhang XK, Van Baalen C, Arnold CR. Feeding and reproductive performance of the harpacticoid *Tisbe carolinensis* (Copepoda, Crustacea) in four algal cultures. Mar. Ecol. Prog. Ser. 1985; 24: 273–279.

[48] Huskin I, Anadón R, Alvarez-Marqués F, Harris RP. Ingestion, faecal pellet and egg production rates of *Calanus helgolandicus* feeding coccolithophorid versus noncoccolithophorid diets. J. Exp. Mar. Biol. Ecol. 2000; 248: 239–254. 10771305 10.1016/s0022-0981(00)00167-2

[49] Kiørboe T, Saiz E, Viitasalo M. Prey switching behaviour in the planktonic copepod Acartia tonsa. 1996; Mar. Ecol. Prog. Ser. 143: 65–75.

[50] Saage A, Vadstein O, Sommer U. Feeding behaviour of adult *Centropages hamatus* (Copepoda, Calanoida): Functional response and selective feeding experiments. 2009; J. Sea Res. 62: 16–21.

[51] Jackson G. A model of the formation of marine algal flocs by physical coagulation processes. 1990; Deep-Sea Res. 37: 1197–1211.

[52] Paffenhöfer GA. Oxygen consumption in relation to motion of marine planktonic copepods. Mar. Ecol. Prog. Ser. 2006; 317:187–192.

[53] Herman PMJ, Heip C. The respiration of five brackish-water harpacticoid copepod species. J. Exp. Mar. Biol. Ecol. 1983; 71: 249–256.

[54] Gaudy R. Etude de la respiration chez des copépodes pélagiques méditerranéens (bassin occidental et Mer Ionienne) et de ses variations en fonction de la bathymétrie des espèces et de leur origine géographique. Mar. Biol. 1975; 29: 109–118.

[55] Halsband C, Hirche H-J. The reproductive cycles of dominant calanoid copepods in the North Sea. Mar. Ecol. Prog. Ser. 2001; 209: 219–229.

[56] Straile D. Gross growth efficiencies of protozoan and metazoan zooplankton and their dependence on food concentration, predator-prey weight ratio and taxonomic group. Limnol. Oceanogr. 1997; 42: 1375–1385.

[57] Alldredge AL. Silver MW. Characteristics, dynamics and significance of marine snow. Prog. Oceanogr. 1988; 20: 41–82.

[58] Böttger-Schnack R. Summer distribution of micro- and small mesozooplankton in the Red Sea and Gulf of Aden, with special reference to non-calanoid copepods. Mar. Ecol. Prog. Ser. 1995; 118: 81–102.

[59] Paffenhöfer GA, Mazzocchi MG. Vertical distribution of subtropical epiplanktonic copepods. J. Plankton Res. 2003; 25:1139–1156.

[60] Arendt KE, Juul-Pedersen TA, Mortensen J, Blicher M, Rysgaard S. A five years study of seasonal patterns in mesozooplankton community structure in a sub-arctic fjord reveals dominance of *Microsetella norvegica* (Crustacea, Copeoda). J. Plankton Res. 2013; 35: 105–120.

[61] Wassmann P, Duarte CM, Agustí S, Sejr MK. Footprints of climate change in Arctic marine ecosystem. Global Change Biol. 2011; 17: 1235–1249.57.

[62] Broekhuizen N, McKenzie E. Patterns of abundance for *Calanus* and smaller copepods in the North Sea: time-series decomposition of two CPR data sets. Mar.Ecol. Prog. Ser. 1995; 118: 103–120.

[63] Beaugrand G, Reid PC, Ibanez F, Lindley JA, Edwards M. Reorganization of North Atlantic marine copepod biodiversity and climate. Science 2002; 296: 1692–1694. doi: 10.1126/science.1071329 12040196 10.1126/science.1071329

[64] Giering SLC, Sanders R, Martin AP, Lindemann C, Möller K, Daniels CJ, et al High export via small particles before the onset of the North Atlantic spring bloom. J. Geophys. Res. Oceans 2016; 121: 6929–6945.

[65] Castellani C, Robinson C, Smith T, Lampitt RS. Temperature affects respiration rate of *Oithona similis*. Mar. Ecol. Prog. Ser. 2005; 285: 129–135.

