# Supplementary figures and images for: Feeding on dispersed vs. aggregated particles: The effect of zooplankton feeding behavior on vertical flux
Source: PLoS One. 2017 May 17;12(5):e0177958. doi: 10.1371/journal.pone.0177958 (PMC5435449; doi:10.1371/journal.pone.0177958)

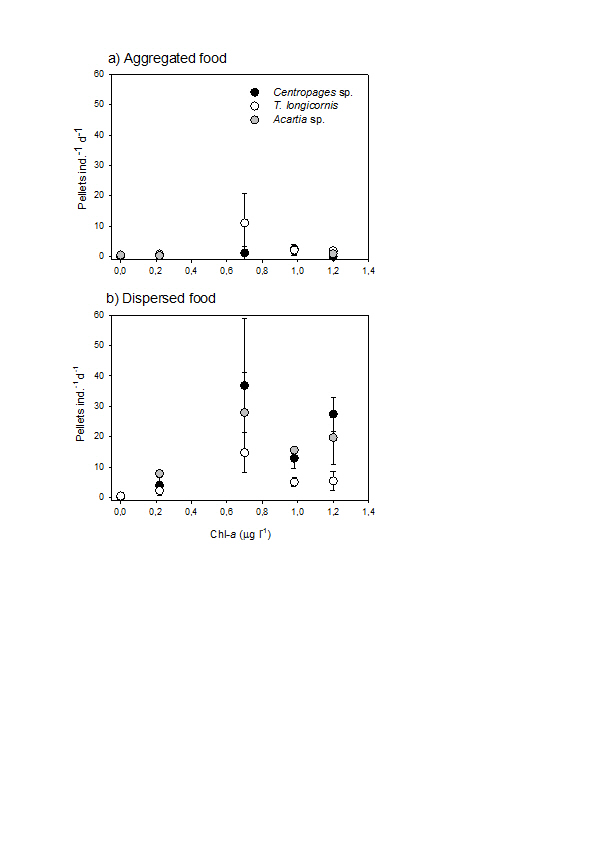

Supplement: S1 Fig — Pellet production (pellets ind.-1 d-1) of Centropages sp., Temora longicornis and Acartia sp. as a function of Chl-a concentration (μg l-1) in mesocosms media in a) aggregated and b) dispersed treatments (mean ± SD). (Closed circles) Centropages sp., (open circles) T. longicornis, (grey circles) Acartia sp. (JPG) [file pone.0177958.s001.JPG]
